# Plant-Derived Lectins as Potential Cancer Therapeutics and Diagnostic Tools

**DOI:** 10.1155/2020/1631394

**Published:** 2020-05-15

**Authors:** Milena Mazalovska, J. Calvin Kouokam

**Affiliations:** ^1^Department of Pharmacology and Toxicology, University of Louisville School of Medicine, University of Louisville, Louisville, KY 40202, USA; ^2^Center for Predictive Medicine, University of Louisville, Louisville, KY 40202, USA; ^3^James Graham Brown Cancer Center, University of Louisville School of Medicine, Louisville, KY 40202, USA

## Abstract

Cancer remains a global health challenge, with high morbidity and mortality, despite the recent advances in diagnosis and treatment. Multiple compounds assessed as novel potential anticancer drugs derive from natural sources, including microorganisms, plants, and animals. Lectins, a group of highly diverse proteins of nonimmune origin with carbohydrate-binding abilities, have been detected in virtually all kingdoms of life. These proteins can interact with free and/or cell surface oligosaccharides and might differentially bind cancer cells, since malignant transformation is tightly associated with altered cell surface glycans. Therefore, lectins could represent a valuable tool for cancer diagnosis and be developed as anticancer therapeutics. Indeed, several plant lectins exert cytotoxic effects mainly by inducing apoptotic and autophagic pathways in malignant cells. This review summarizes the current knowledge regarding the basis for the use of lectins in cancer diagnosis and therapy, providing a few examples of plant-derived carbohydrate-binding proteins with demonstrated antitumor effects.

## 1. Introduction

Cancer is considered a serious threat to human health globally, constituting the second most frequently diagnosed and deadliest pathology after cardiovascular diseases among ailments of noninfectious etiology [1]. Indeed, the incidence and death rates are steadily rising worldwide, with above 18 million new cases and approximately 10 million deaths caused by malignant diseases [2, 3]. Commonly applied therapeutic options for cancer comprise operation (open surgery or cryoablation), radiotherapy, and chemotherapy [4–6]. These treatment approaches substantially inhibit tumor growth and could even achieve cure, but each has specific advantages and shortcomings.

Chemotherapy is frequently applied for cancer therapy and can be grouped in different categories such as curative (permanent cure following malignant cell elimination), adjuvant (removal of residual undetectable cancer cells following surgery), neoadjuvant (preoperative lesion shrinking), and palliative (symptom alleviation, reduction of complications) types [7, 8]. In general, chemotherapeutics suppress target cells by modulating distinct molecules in various pathways of rapidly growing malignant cells but unfortunately exert deleterious effects on noncancerous cells, with multiple and sometimes serious adverse events [9]. Therefore, developing novel and more selective agents that could target and distinguish malignant cells would likely improve cancer patient prognosis. Meanwhile, lectins display differential binding patterns to cancerous tissues according to the extent of glycosylation and could therefore be employed not only as diagnostic tools but also as anticancer agents [10, 11].

Lectins are ubiquitously found in bacteria, fungi, plants, and animals [12–14]. The term lectin was coined by Boyd and Shapleigh in 1954, to indicate a nonimmunoglobulin protein that binds carbohydrate molecules without modifying them [15]. Lectins are currently considered carbohydrate-binding proteins that reversibly interact with specific saccharides in glycoproteins and glycolipids [16]. Lectins differ by their biophysiochemical properties, inhibiting various organisms, including fungi, viruses, and insects, while also acting as immunomodulatory molecules [17–19]. Additionally, lectins are involved in immune defense, cell migration, cell-to-cell interactions, embryogenesis, organ formation, and inflammation [20, 21].

Lectins protect plants from insects and fungi and are also involved in sugar transport and storage [22]. In addition, some lectins are critical for atmospheric nitrogen fixation [22]. Based on overall structure and the number of carbohydrate binding domains, plant lectins are grouped into hololectins, chimerolectins, superlectins, and merolectins [23]. Additionally, they comprise 12 distinct families that show diverse carbohydrate-binding specificities, including Agaricus bisporus agglutinins, Amaranthins, class V chitinase homologs with lectin activity, Cyanovirins, EEA lectins, GNA lectins, Heveins, Jacalin-related lectins, legume lectins, LysM lectins, Nictaba lectins, and Ricin B lectins [24]. Of these, legume lectins, Ricin B proteins, and GNA-related lectins constitute the most investigated classes, due to remarkable biological functions. Recently, plant lectins have attracted growing attention for selectively and sensitively targeting cell surface glycans, with potential applications in multiple fields [24–27]. This review provides a theoretical basis for applying lectins in cancer diagnosis and therapy, listing a few examples that demonstrate antiproliferative and anticancer activities via autophagy and apoptosis.

## 2. The Glycocalyx of Eukaryotic Cells

**Figure 1:**
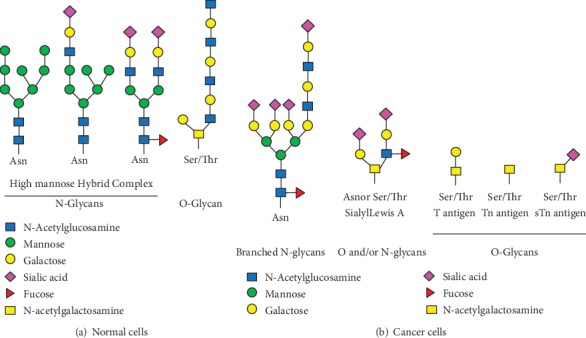
Schematic representation of select N- and O-glycans found in normal and cancer cells. (a) Normal cells have three major types of N-glycans, including high mannose, hybrid, and complex types. The precursor unit is added to the protein through an N-glycosidic bond with the side chain of an asparagine residue that is part of the Asn-X-Ser/Thr consensus sequence. The precursor is trimmed, with additional residues added in the Golgi complex. The first step in O-linked glycosylation involves N-acetylgalactosamine addition to a serine or threonine residue of the polypeptide chain that can proceed with adding other monosaccharides such as galactose, fucose, and sialic acid. (b) Cancer cells have altered glycosylation patterns, comprising either production of new glycans or incomplete synthesis of original glycans. The most common change in N-linked glycoproteins is the production of branched N-glycans; sialyl Lewis A antigen is found in both N- and O-linked, while Tn, sTn, and T antigens are found in O-linked glycoproteins. Glycan structures were adapted from *Essentials of Glycobiology* 3^rd^ edition [29].

The cell surface in eukaryotes encompasses proteins and carbohydrates, which constitute the glycocalyx [28]. Glycans are very diverse and complex, with various monosaccharides, glycan attachment sites, and bond and branching types [29]. They are mostly linked to the nitrogen of asparagine moieties (N-glycans) or through the oxygen of serine or threonine moieties (O-glycans) on secreted or membrane-bound glycoproteins, with a broad range of structures (Figure 1(a)). Glycans contribute to protein folding, cell-to-cell interactions, pathogen recognition, immune reactions, antigen presentation, and cell adhesion and migration, thereby affecting multiple cellular processes [30–33]. Mature glycoproteins vary N-linked oligosaccharides according to cell type, tissue, and species [34]. All eukaryotic cells share the basic mechanisms of glycoprotein synthesis; however, marked differences exist between malignant and noncancerous cells.

## 3. Differences in Glycosylation Patterns between Malignant and Noncancerous Cells

Abnormal glycosylation, with either novel glycoforms formed or incompletely synthesized glycans, represents an important hallmark of malignancy [30]. Indeed, altered glycosylation patterns generate multiple biomarkers, some of which are associated with cell malignancy [30]. N-Linked glycoproteins' alterations mostly comprise the generation of branched oligosaccharides. For example, increased amounts of N-acetylglucosaminyltransferase V (MGAT5) induces the *β*1-6Glc-NAc branching of glycoproteins; this modification contributes to malignancy [35]. Additionally, elevated *β*1-6Glc-NAc branching of N-linked oligosaccharides promotes sialylation, which is involved in cancer metastasis [36]. It is known that O-linked T, Tn, and sTn antigens are all expressed in multiple malignancies such as breast, lung, colon, cervical, and bladder cancers, but absent in noncancerous cells or tissues (Figure 1(b)) [37]. Many lesions coexpress modified O-glycans, whose upregulation could help predict poor prognosis in some cancers [38, 39]. Altered terminal residues are also related to malignancy, with enzymes catalyzing their addition overexpressed in malignant cells [38]. Examples comprise sialyl Lewis X, sialyl-Tn, Thomas Friedrich (TF), Globo H, Lewis Y, and polysialic acid (PSA) [39]. Overexpression of sialyl- Lewis A/X antigen is shared by many epithelial malignancies (Figure 1(b)); this alteration is associated with loss of ABH blood antigens and poor prognosis [40]. Other glycoproteins and glycolipids show a trend of overproduction in cancer, e.g., gangliosides and mucins. For instance, elevated ganglioside amounts are found in head and neck tumors, neuroblastoma, melanoma, medulloblastoma, lung cancer, and breast cancer [41]. Growing evidence demonstrates that altered glycosylation affects the malignancy potential, tumor immune surveillance, and prognosis and could be employed to develop novel tools for cancer diagnosis and therapy [42].

## 4. Lectins as Potential Diagnostic Tools

**Figure 2:**
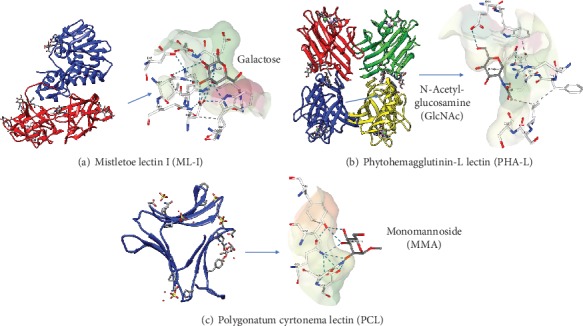
Crystal structures of three representative lectins showing their interactions with the corresponding sugars via carbohydrate-binding domains (CBDs). (a) Mistletoe lectin I (Ricin family) with A chain (blue) and B chain (red) shown as a ribbon in a complex with galactose (protein data bank [PDB]: 1OQL). (b) The tetrameric phytohemagglutinin-L (legume family) with monomers in various colors, in complex with GlcNAc (PBD: 1FAT). (c) *Polygonatum cyrtonema* (GNA-related family) as a monomeric protein in complex with monomannoside (PDB: 3A0D). Dotted lines are hydrogen bonds. The structures were generated with the UCSF Chimera software (Resource for Biocomputing, Visualization, and Informatics (RBVI), USA). Binding of lectins to their respective sugars used the PDB.

Lectin interactions with target ligands involve a vast array of hydrogen bonds, as well as hydrophobic and nonpolar van der Waals interactions. Small structural alterations of the carbohydrate-binding domains might lead to great differences in ligand binding, with important modifications of biological functions. For instance, PCL and other GNA-related lectins have three putative sugar-binding sites (CBD I, CBD II, and CBD III). Polar amino acids (Gln, Asp, and Tyr) engage in hydrogen bonds with O_2_, O_3_, and O_4_ of mannose while Val interaction with mannose involves hydrophobic interactions. The crystal structure of PLC revealed CBD I as the major mannose-binding domain, while CBD II in which Gln58 and Asp60 are replaced by His58 and Asn60, respectively, no longer interacts with mannose (Figure 2(c)) [43]. Although CBD III has mannose-binding features in other GNA-related lectins, CBD III in PCL instead interacts with other ligands, e.g., sialic acid [44]. These distinct binding features of PCL could explain its biological functions in inhibiting viruses and cancer.

Since various lectins interact with terminal sugars on glycoproteins and glycolipids with high specificity, they could help characterize cell surface alterations in cancer. To this end, wheat germ agglutinin (WGA) was the first lectin shown to agglutinate cancer cells, indicating modified cell surface properties in malignant cells compared with noncancerous ones [45]. Similarly, phytohemagglutinin (PHA-L) produced by *Phaseolus vulgaris*, which is specific to complex-type N-glycans, helps detect modified N-linked carbohydrate core structure or *β*1-6-GlcNac moiety that is associated with malignancy in colon and pancreatic cancers (Figure 2(b)) [46, 47]. In addition, *Lens culinaris* agglutinin (LCA) from lentil seeds with *α*1-6 fucose specificity could be employed in early hepatocellular carcinoma (HCC) diagnosis through interaction with the HCC marker *α* fetoprotein (AFT). Furthermore, the fucosylated *α* fetoprotein-L3 (AFTL3) isoform shows higher specificity in HCC diagnosis in comparison with total AFT and worsens prognosis [48]. The lectin LCA also interacts with serum thyroglobulin (Tg), a biomarker of thyroid cancer, distinguishing between noncancerous and malignant diseases [49]. Moreover, LCA and *Aleuria aurantia* lectin (AAL) bind to fucosylated prostate-specific antigen (PSA), a commonly employed early diagnostic marker of prostate cancer [50, 51].

Many other plant lectins could help detect malignant tumors. *Wisteria floribunda* agglutinin (WFA), a legume lectin, preferentially interacts with glycans possessing terminal N-acetylgalactosamine. WFA has high affinity to the cancer biomarker L1 cell adhesion molecule (L1CAM) and has been employed for detecting intrahepatic cholangiocarcinoma (CC) [52]. In addition, WFA is lowly specific to another highly glycosylated biomarker, mucin 1 (MUC1) [53]. However, it was suggested that combining both biomarkers would improve diagnostic accuracy and reliability in CC [52].

The current diagnostic biomarker of ovarian cancer is the human cancer antigen CA125; however, serum CA125 amounts are also elevated in some nonmalignant pathologies [54, 55]. Because CA125 is unreliable for diagnosing ovarian cancer, Shewell et al. suggested N-acetylneuraminic acid, which is a major form of glycosylation on cancer cells [56]. Additionally, glycans with terminal N-glycolylneuraminic acid are not detected in considerable amounts in nondiseased tissues in humans. The latter research team engineered the lectin SubB2M based on the B subunit of Shiga toxigenic *E. coli* Subtilase cytotoxin (SubAB) with ameliorated Neu5Gc glycan recognition, which was capable of detecting high serum amounts of Neu5Gc-glycans in patients with ovarian cancer (all stages) versus noncancerous controls [56].

*Ricinus communis* agglutinin I (RCA-I), which specifically interacts with terminal galactose moieties, binds the membrane glycoprotein POTE ankyrin domain family member F (POTEF) in triple-negative breast cancer (TNBC) cells proportionally to their metastatic abilities [10]. Hence, RCA-1 might help diagnose TNBC and predict the odds of metastasis in TNBC cases.

Recently, the galactose-specific lectins *Aleuria aurantia* lectin (AAL), *Ulex europaeus* I (UEA-I) fucose-binding lectin, *Agaricus bisporus* agglutinin (ABA), *Maclura pomifera* (MPL), and Phaseolus vulgaris erythroagglutinin (PHA-E) were shown to differentiate between metastatic and nonmetastatic pancreatic cancer cells [57]. The same team demonstrated that genes encoding fucosyltransferases and altering N-linked fucosylation are overexpressed in metastatic pancreatic cancer, providing a possible mechanism by which AAL also blunts motility in metastatic pancreatic cancer. The above examples suggest that lectins might be useful in detecting tumor markers and differentiating between cancer and nonmalignant cells through targeting of specific differentially glycosylated isoforms.

## 5. Lectins Inhibit Cancer Cells Mostly by Inducing Autophagy and Apoptosis

Understanding how plant lectins exert antiproliferative and cytotoxic effects on cancer cells might help develop novel potent anticancer agents. Previous reports have revealed the ability of plant lectins to bind to tumor cell surface and promote apoptotic and autophagic cell death [27, 58]. Representative lectins are described below, which promote cell death by modulating apoptotic and autophagic signaling pathways (Table 1).

### 5.1. Ricin B Family of Proteins with Cancer Inhibitory Properties

One of the most toxic plant protein groups is the Ricin B family of ribosome-inactivating proteins (RIPs). RIPs attract increasing interest for their reported therapeutic potential as well as possible utilization in biological warfare and bioterrorism [59]. Plant RIPs comprise two major classes, including types I (single-chain molecules with enzymatic activity) and II (an enzymatic A subunit and one or many B chains with lectin activity) [60]. Since the B subunit has affinity for cell surface sugar-containing molecules, it is involved in the translocation of the toxic A chain into the cytosol where it inhibits ribosomes. RIPs specifically and irreversibly suppress protein synthesis in eukaryotes by enzymatically altering the 28S rRNA of the 60S ribosomal subunit [58]. Plant RIPs comprise ricin, abrin, mistletoe lectins, Korean mistletoe lectin, modeccin, and volkensin, some of which are briefly discussed below [61–64].

#### 5.1.1. Mistletoe Lectins

**Figure 3:**
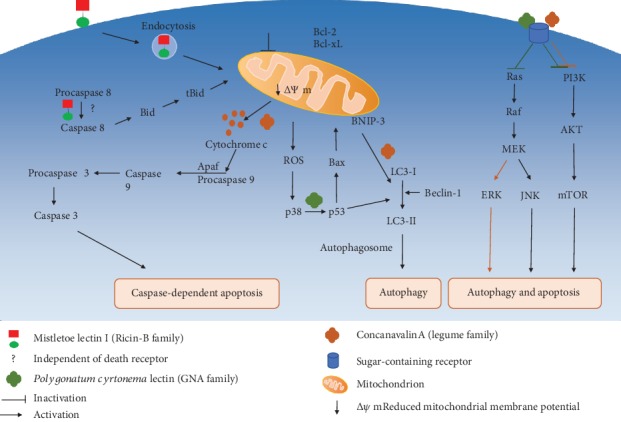
Signaling pathways of selected plant lectins involved in apoptosis and/or autophagy.

Mistletoe lectins (type II RIPs) are arguably the most investigated proteins for cancer treatment. European mistletoe (*Viscum album*) represents a semiparasitic plant growing on multiple trees in Europe, Asia, and North Africa [65]. For centuries, *V. album* extracts have been traditionally applied for treating diverse ailments including seizures, hypertension, wounds, and headaches [66, 67]. The compositions of such preparations vary by host tree, extraction method, and harvest season [68]. Currently, standardized aqueous mistletoe extracts are common in complementary and alternative medicine (CAM) for treating nonmalignant and cancerous lesions in humans, particularly in Europe [69, 70]. Multiple constituents showing cytotoxic and immunomodulatory properties, as well as potential anticancer effects, have been detected in mistletoe extracts, including viscotoxins, polysaccharides, lectins, phenolic acids and flavonoids, triterpenes, and polypeptides [71, 72]. However, mistletoe lectins (ML-I, II, and III) are considered the major constituents with antitumoral features [63, 73]. ML-I to III possess cytotoxic (A) and carbohydrate-binding (B) subunits, linked by disulfide bonds to form heterodimers [63]. However, they have differing sugar-binding specificities: ML-I interacts with D-galactose, ML-III with N-acetyl-galactosamine, and ML-II with both (Figure 2(a)) [74]. As RIPs, MLs bind cells via the B subunit, delivering the toxic A subunit into the cytoplasm [62, 75]. Upon internalization, the A subunit suppresses protein synthesis in eukaryotes via hydrolysis of the N-glycosidic bond linking adenine-4324 and G-4325 in 28S rRNA. This rRNA depurination renders the ribosome unable to bind cell factors, consequently blunting protein production [62]. New evidence suggests that the potent cytotoxicity of ML-I in malignant cells is not only due to rRNA depurination but also to the A chain affecting multiple other adenine-containing substrates [76, 77]. It was demonstrated that ML-1 as well as its B chain alone have immunostimulatory features, upregulating cytokines such as TNF-*α*, interleukin- (IL-) 1, IL-6, and granulocyte macrophage colony-stimulating factor (GM-CSF) upon treatment of peripheral blood mononuclear cells (PBMCs) with ML-I [78]. Such cytokine upregulation is also present in cancer cases treated with mistletoe extracts with adequate ML-I amounts, indicating that mistletoe lectin-carbohydrate interactions induce nonspecific defense pathways that could benefit cancer treatment [78]. The above research team also reported higher amounts of large granular lymphocytes and NK killers upon parenteral injection of rabbits with low doses of ML-I and the related B chain, respectively [79]. NK cells as effector cells contribute to cancer inhibition, by carrying out immune surveillance against primary tumors and preventing cancer spread [80]. However, the major activity of ML-I is its cytostatic and cytotoxic effects on various cancer cell lines. It is widely admitted that ML-I's cytotoxic effects are mediated by apoptosis induction. Treatment of leukemic T and B cells with ML-I results in caspase-8/FLICE, caspase-9, and caspase-3 activation, leading to apoptotic cell death (Figure 3) [81]. Meanwhile, it was determined that ML-I uptake in CT26 mouse colon carcinoma cells is energy dependent, with translocation into the cell occurring by both clathrin-dependent and clathrin-independent mechanisms. After uptake, ML-I is redirected towards the Golgi, undergoes retrograde transport to the ER, and translocates to the cytoplasm in a manner similar to ricin. It was recently shown that ML-I's proapoptotic activity is caspase mediated [82]. On the other hand, the antiproliferative effects of ML-I differ according to the cancer cell lines, which might be related to varying cell glycosylation patterns [82]. Interestingly, it was demonstrated that a mistletoe extract or recombinant ML-I alone could induce NK-cell killing of glioma. Additionally, ML-I enhances the antiglioma effects of T cells as well as animal survival when jointly administered with chemotherapeutics in the mouse model of glioma, suggesting potential adjuvant effects [83]. *V. album* preparations have been assessed clinically for antitumor features, alone or in combination with current treatments; however, their mode of action and therapeutic benefits remain largely undefined. Clinical trials evaluating mistletoe extracts reported a decrease of adverse events due to conventional cancer therapies, with enhanced survival and no adverse interactions with the antitumor agents being applied [84, 85]. Others, however, do not strongly support ML extracts as antitumor products or adjuvant therapeutics in cancer [86].

#### 5.1.2. Korean Mistletoe Lectin

Korean mistletoe lectin, produced by *Viscum album* coloratum (KMLC), interacts with galactose and N-acetylgalactosamine and induces apoptosis in cancer cells while exerting immunomodulatory effects via NK cell induction; KMLC's antitumor properties result from enhanced NK cell cytotoxicity via perforin upregulation [87]. KMLC also induces apoptotic pathways in two hepatoma cancer cells, including SK-Hep-1 (p53 positive) and Hep3B (p53 negative) cells, independent of p53 and p21 signaling cascades. Indeed, this lectin promotes apoptosis by inducing Bax and suppressing Bcl-2, subsequently activating caspase-3 [88]. Others also reported that apoptosis-related cell death in Hep3B cells is caused by ROS generation, low mitochondrial membrane potential, cytochrome c release in the cytosol, and SEK/JNK pathway induction [89]. Further, it was demonstrated that apoptosis is triggered in KMLC-treated A253 cells through suppression of telomerase activity, reduced Akt phosphorylation, and caspase-3 induction [90].

### 5.2. Legume Lectins with Anticancer Properties

Legume lectins constitute a large group of homologous carbohydrate-binding proteins. They feature two or four equivalent subunits and are specific to multiple sugars, from monosaccharides to complex carbohydrate molecules [107]. They mostly encompass mannose/glucose-specific (e.g., Con A) and galactose/N-acetylgalactosamine-specific (e.g., *Bauhinia forficata* lectin (BFL)) types.

#### 5.2.1. Concanavalin A

Concanavalin A (Con A), a well-known protein with mannose/glucose specificity, was the first lectin purified from Jack bean seeds a century ago. Its antiproliferative effects on human melanoma A375 cells involve caspase-related apoptosis [108]. Additionally, Con A promotes autophagic pathways in HeLa cells through Pl3K/Akt/mTOR pathway suppression and MEK/ERK axis upregulation (Figure 3) [92]. Moreover, low Con A levels activate innate immune cells in the liver and exert inhibitory effects on Colon-26 cancer cells in mouse models [109]. It is admitted that Con A's effects are mediated by internalization and targeting to the mitochondria, resulting in hepatoma cell autophagy; according to treatment time, dose, and frequency, Con A could display anticancer properties in hepatoma-bearing SCID mice [94]. In addition, Con A administration to glioblastoma U87 cells upregulates BNIP3 and autophagy-associated genes, providing a basis for an autophagic mechanism of action for Con A (Figure 3) [93].

#### 5.2.2. Dioclea Violacea Lectin

*Dioclea violacea* lectin (DVL) produced by *Dioclea violacea* seeds is another legume protein showing mannose/glucose specificity and anticancer features [110]. DVL enhances caspase-3 activation and apoptotic cell death in rat glioma C6 cells [95]. Further, DVL was shown to inhibit U87 cells via autophagy enhancement by suppressing effectors such as Akt, ERK1/2, and TORC1, which are overexpressed in cancer [96].

#### 5.2.3. *Dioclea lasiocarpa* Lectin

*Dioclea lasiocarpa* lectin (DLL) represents a mannose/glucose-specific lectin produced by *Dioclea lasiocarpa* Mart seeds [111]. DLL exerts strong antiproliferative effects on various cancer cells such as A549, MCF-7, PC3, and A2780 cells, of which ovarian cancer A2780 cells were most susceptible with elevated high mannose content on the cell surface [112, 113]. In addition, DLL suppresses glioma cell lines by inducing caspase-3 activation, autophagy, and cell death [97].

#### 5.2.4. *Dioclea lasiophylla* Lectin

*Dioclea lasiophylla* lectin (DlyL) is a mannose-specific carbohydrate-binding protein obtained from *Dioclea lasiophylla* Mart. Ex Benth seeds. It has a high affinity towards N-glycans of the complex or hybrid types. It was also shown to exert antitumor effects on rat glioma C6 cells, inhibiting cell migration and inducing autophagy and cell death via caspase-3 activation [98].

#### 5.2.5. Legume Lectins from *Bauhinia* spp.

Another legume lectin, *Bauhinia forficata* lectin (BFL), produced by *B. forficata* with specific interaction with GalNac, is toxic to cultured breast cancer MVF7 cells. BFL exerts cytotoxicity via caspase-9 suppression and subsequent G2/M phase arrest [99]. Additionally, BFL inhibits various malignant cells in the NCI-60 panel, with melanoma LOX IMVI cells showing highest susceptibility [114]. Two other lectins, *Bauhinia variegata* lectin (BVL) and *Bauhinia ungulate* lectin (BUL) detected in *Bauhinia* spp., have demonstrated antitumor properties. BLV displays low-micromolar growth suppression of breast cancer MCF7 and hepatoma HepG2 cells [115], while BUL is dose-dependently cytostatic in colon adenocarcinoma HT-29 cells [116].

Mistletoe lectin I triggers caspase activation and apoptosis via a death receptor-independent, but mitochondria-mediated pathway in leukemic T and B cells. ML-1 internalization occurs by endocytosis, which is critical for the lectin's cytotoxicity. This is followed by cytochrome c release and mitochondrial membrane potential reduction, inducing the caspase cascade which causes caspase-associated apoptosis. Treatment with ML-1 also enhances caspase-8 induction, without involving death receptors [82]. Another lectin, *Polygonatum cyrtonema*, triggers both autophagy and apoptosis in cancer. PCL interacts with sugar-containing receptors on cells and induces autophagy by inhibiting the PI3K/AKT/mTOR and Ras-Raf pathways in murine fibrosarcoma L929 cells. Culture of another cancer cell line, A375 cells, in the presence of PCL induces autophagic and apoptotic cell death via mitochondria-associated ROS-p38-p53 pathway. In HeLa cells, Concanavalin A (Con A) suppresses PI3K/AKT/mTOR signaling and induces the MEK/ERK pathway, resulting in autophagy. In hepatoma cells, Con A triggers autophagy via mitochondria-mediated pathway [92]. Additionally, Con A administration to A375 cells inhibits them through caspase-associated apoptosis [93].

### 5.3. GNA-Related Lectin Family Members with Antitumoral Features

*Galanthus nivalis* agglutinin- (GNA-) related lectins constitute a superfamily of strictly mannose-binding proteins active against cancer, viruses, and fungi [117]. The first GNA-related lectin, coined *Galanthus nivalis* lectin (or GNA), was obtained from snowdrop bulbs [118, 119]. GNA-related lectins were previously renamed “monocot mannose-binding lectins”. However, after isolating and characterizing many other lectins with GNA domains, they are currently referred to as GNA-related lectins [118].

#### 5.3.1. *Polygonatum cyrtonema* Lectin

*Polygonatum cyrtonema* lectin (PCL), a mannose/sialic acid-binding lectin, induces apoptotic and autophagic death in human melanoma A375 cells; apoptosis induction involves Bax and Bcl-2 regulation at the protein level, which remarkably reduces the mitochondrial membrane potential, with subsequent cytochrome c release and caspase activation [100]. Additionally, PCL enhances ROS production as well as p38 and p53 activation, which contribute to autophagy in A375 cells, suggesting that PCL triggers both cell death mechanisms simultaneously [100]. PCL also triggers autophagy and apoptosis in mouse fibrosarcoma L929 cells, via Ras-Raf and Pl3K-Akt signaling suppression (Figure 3) [101]. Recent evidence suggests that PCL enhances autophagic and apoptotic death in A549 cells through ROS-dependent MAPK and NF-*κ*B pathway regulation [102]. Further, PCL and two other prototypic GNA-related lectins, i.e., *Ophiopogon japonicus* lectin (OJL) and *Liparis nervosa* lectin (LNL), exert suppressive effects on MCF-7 cells, enhancing caspase-dependent apoptosis [103].

#### 5.3.2. *Polygonatum odoratum* Lectin

*Polygonatum odoratum* lectin (POL) produced by *Polygonatum odoratum* (Mill.) Druce also represents a GNA-related lectin that enhances caspase-associated apoptosis in A375 and L929 cells [103, 104]. POL was demonstrated to simultaneously trigger apoptotic and autophagic death in A549 cells; apoptosis and autophagy were enhanced by the Akt-NF-*κ*B pathway and Akt-mTOR signaling suppression, respectively [105].

#### 5.3.3. *Remusatia vivipara* Lectin

*Remusatia vivipara* lectin (RVL) is another mannose-binding protein produced by *Remusatia vivipara* (Araceae), with potent nematicidal activity [120]. RVL shows high affinity to N-linked glycans, but no interactions with O-linked glycans and monosaccharides. It exerts inhibitory and anticell migratory effects on breast cancer MDA-MB-468 and MCF-7 cells, via apoptosis [106].

## 6. Conclusions and Perspectives

Plant lectins attract wide attention owing to their multifaceted properties in agriculture, blood typing, and diagnosis. In the past decade, a decent number of lectins have been purified with diverse carbohydrate specificities, providing novel directions in lectin research. Currently, lectins are broadly employed in glycobiology research, e.g., for detecting and analyzing glycoproteins, carbohydrate assessment on cells, cell identification and isolation/separation, immunohistochemical analysis, bacterial typing, mapping central neuronal pathways, diagnosis, and tracing [121, 122]. Lectins such as Con A are routinely applied to isolate glycoproteins by affinity chromatography [123]. Lectin-based microarrays help assess the structural alterations of glycans and enable screening and assessment of the glycosylation profiles of therapeutic proteins [124].

In the pharmaceutical field, lectins constitute potential antitumor therapeutics. Specifically, they can differentiate between noncancerous and malignant lesions. Indeed, multiple lectins demonstrate anticancer features in cultured cells and in vivo, with some inhibiting malignant cells via apoptosis and/or autophagy by regulating multiple pathways. Mistletoe extracts are extensively applied for cancer treatment in Europe, with antineoplastic and apoptosis-inducing properties, as well as immunostimulatory and antiangiogenic features [125, 126].

Despite the anticancer features of lectins, there are few drawbacks hindering their development for cancer therapy. A potential issue is toxicity. For example, Con A induces liver failure upon intravenous administration in mouse models [127]. In addition, PHA-L causes nausea, vomiting, and diarrhea following oral treatment [128]. Furthermore, some RIPs (e.g., abrin and ricin) are toxic to mice with low LD50s of 10-13 and 55-65 ng, respectively [129]. Such toxicity could be mitigated by fusing lectins to other proteins for targeted delivery. For example, immunotoxins were designed for selective delivery of a toxin to malignant cells by linking a toxic domain to a specific targeting moiety, e.g., an antibody, or the Fab, Fc, or single-chain variable fragments [130]. Ricin has been widely assessed for such purpose [131]. Another chimeric molecule with antiviral activity, Avaren-Fc, designed by fusing the lectin actinohivin to the Fc region of human immunoglobulin G1, demonstrates potential anticancer activity [132].

Future research aspects of developing lectins as anticancer agents should involve in vitro and in vivo assessments of immunomodulatory and toxic effects on healthy cells. In addition, further understanding of their mechanisms of action and roles in autophagy and apoptosis-induced cell death could provide better targets for cancer therapy in the future.

## Figures and Tables

**Table 1:** Representative plant lectins that induce cell death by modulating apoptotic or autophagic signaling pathways.

Lectin	Sugar(s) bound	Cancer type(s) and/or cancer cell line(s)	Mechanism(s) of cell growth inhibition/target molecule(s) or pathway(s)	References
Mistletoe lectin I (ML-I)	Galactose	Leukemic T and M cells	Apoptosis/activation of caspase-8/FLICE, caspase-9, and caspase-3	[81]
		CT26 cells	Apoptosis/ROS generation and activation of SEK/JNK pathway	[82]
		Glioma (in mice)	Apoptosis/caspase-dependent pathway, activation of NK cells	[83]

Korean mistletoe (KMLC)	Galactose/N-acetylgalactosamine	SK-Hep-1 cells/Hep3B cells	Apoptosis via p21- and p53-independent pathways/activation of Bax and caspase-3, inhibition of Bcl-2	[88]
		Hep3B	Apoptosis/ROS generation and activation of SEK/JNK pathway	[89]
		A253 cells	Apoptosis/inhibition of telomerase activity, decreased phosphorylation of Akt, and activation of caspase-3	[90]

Concanavalin A (Con A)	Mannose/glucose	A375 cells	Apoptosis/caspase-dependent manner	[91]
		HeLa cells	Autophagy/suppressing Pl3K/Akt/mTOR and upregulating MEK/ERK	[92]
		U87 cells	Autophagy/upregulation of BNIP3	[93]
		Hepatoma (in SCID mice)	Antitumor effect	[94]

*Dioclea violacea* (DVL)	Mannose/glucose	Rat C6 glioma cells	Apoptosis/caspase-3 activation	[95]
		U78 cells	Autophagy/inhibition of Akt, ERK1/2, and TORC1	[96]

*Dioclea lasiocarpa* lectin (DLL)	Mannose/glucose	A549, MCF-7, PC3, A2780, glioma cell lines	Induction of autophagy/activation of caspase-3	[97]

*Dioclea lasiophylla* lectin (DlyL)	Mannose	Rat C6 glioma cells	Induction of autophagy/activation of caspase-3	[98]

*Bauhinia forficata* (BFL)	N-Acetylgalactosamine	MVF7 cells	Apoptosis/inhibition of caspase-9	[99]

*Polygonatum cyrtonema* lectin (PCL)	Mannose/sialic acid	A375 cells	Apoptosis/caspase-activation, ROS accumulation, and activation of p53 and p38	[100]
		L929 cells	Apoptosis and autophagy/through Ras-Raf and Pl3K-Akt signaling pathways	[101]
		A549 cells	Apoptosis and autophagy/ROS-mediated MAPK and NF-*κ*B signaling pathways	[102]
		MCF-7 cells	Apoptosis/caspase-dependent pathways	[103]

*Polygonatum odoratum* lectin (POL)	Mannose	A375 cells	Apoptosis/caspase-dependent	[104]
		L929 cells	Apoptosis/caspase dependent	[103]
		A549 cells	Apoptosis and autophagy/inhibition of Akt-NF-*κ*B or Akt-mTOR pathway	[105]

*Remusatia vivipara* lectin (RVL)	Mannose	MDA-MB-231, MCF-7	Induction of apoptosis	[106]
